# Maternal and paternal harsh parenting and anxiety symptoms in Chinese adolescents: examining a multiple mediation model

**DOI:** 10.1186/s13034-024-00826-9

**Published:** 2024-10-22

**Authors:** Xiujuan Yang, Ling Lin, Wen Feng, Pei Liu, Nana Liang, Zhenpeng Xue, Yuejiao Ma, Yuan Shen, Wenwen Yu, Jianping Lu, Jianbo Liu

**Affiliations:** 1 Shenzhen Mental Health Center, Shenzhen Kangning Hospital, Shenzhen, China; 2 State Key Laboratory of Chemical Oncogenomics, Guangdong Provincial Key Laboratory of Chemical Genomics, Peking University Shenzhen Graduate School, Shenzhen, China; 3 First People’s Hospital of Qingzhen, Qingzhen, China

**Keywords:** Harsh parenting, Anxiety, Self-efficacy, School connectedness, Internet addiction, Sleep problems

## Abstract

**Background:**

Harsh parenting has been recognized as a risk factor for adolescent anxiety; however, the underlying mechanisms of this relationship remain unclear, and it is unknown whether this relationship is influenced by different parental roles and living arrangements. This study aimed to investigate the mediating mechanisms between harsh parenting and adolescent anxiety symptoms using a multiple mediation model and to further compare specific roles of harsh parenting and distinguish between the living arrangements.

**Methods:**

A total of 3505 adolescents completed this survey, and 3295 adolescents (54.7% girls, M_age_ = 14.97 years) were included in the study. Participants completed self-assessments measuring harsh parenting, self-efficacy, school connectedness, Internet addiction, sleep problems, and anxiety. They were categorized into three groups based on living arrangements: living with both parents, only with the mother, or only with the father.

**Results:**

Correlational analyses revealed that both maternal and paternal harsh parenting were associated with increased anxiety symptoms. Structural equation modeling (SEM) mediation analyses and multigroup analyses showed that the independent mediating effects of school connectedness, Internet addiction, and sleep problems, as well as the sequential mediating pathways involving self-efficacy → school connectedness, self-efficacy → Internet addiction, and self-efficacy → sleep problems, vary across the adolescents’ living arrangements in the association between maternal and paternal harsh parenting and adolescent anxiety symptoms.

**Conclusions:**

This study elucidated the mechanisms linking harsh parenting to adolescent anxiety symptoms and validated the effects of different parental roles and living arrangements. The findings provide important insights for developing targeted interventions to address anxiety symptoms in adolescents exposed to harsh parenting.

**Supplementary Information:**

The online version contains supplementary material available at 10.1186/s13034-024-00826-9.

## Introduction

Anxiety is one of the most widely reported mental disorders with far-reaching impacts, leading to functional impairments such as self-injury and increased suicide risk [1]. Anxiety disorders have been recognized as significant contributors to the burden of disease worldwide, particularly among adolescents aged 15 to 24, ranking as the seventh leading cause of disability-adjusted life years [2]. A recent meta-analysis highlighted a concerning trend, revealing a 20.5% global prevalence of clinically elevated anxiety symptoms among children and adolescents during the COVID-19 pandemic [3]. This highlights the importance of focusing on adolescent anxiety and exploring its antecedents and underlying mechanisms.

The unfavorable effects of stress load imbalance on physical and mental health have been well-established [4, 5]. According to the parental acceptance–rejection theory [6], when children perceive rejection, hostility, or indifference from parents or other caregivers, their needs for care and acceptance are thwarted, thus they are prone to a range of psychological problems, such as anxiety symptoms. Empirical studies have found that negative parenting styles, such as abuse, rejection, psychological control, and neglect, serve as chronic stressors contributing to mental health problems among children and adolescents [7–9]. Harsh parenting, characterized by aggressive behaviors from parents, including physical (e.g., corporal punishment) and verbal (e.g., shouting, yelling) forms, is a specific form of negative parenting [10–12]. Numerous studies have identified harsh parenting as a significant risk factor for adolescent anxiety symptoms [13–15]. However, the mechanisms through which harsh parenting influences anxiety in adolescents are still unclear.

The cognitive appraisal theory of stress [16] would serve as the overarching framework to elucidate the mediation processes through which harsh parenting affects adolescent anxiety. This theory posits that the outcomes of stressful situations arise from the interplay between the individual and the environment. When faced with a stressor, individuals cognitively appraise its potential damage or challenge and assess their available resources for efficient coping. This appraisal influences coping strategies. Individuals who believe they can alter adverse conditions are more inclined to adopt problem-focused coping (e.g., redefining problems or seeking alternatives). Conversely, those who perceive themselves unable to alter their circumstances tend to adopt emotion-focused strategies (e.g., avoidance or distancing). These coping strategies are linked to social functioning, behavioral adaptation, and physical health, ultimately affecting mental health outcomes. This study posits that harsh parenting serves as a familial stressor impacting adolescent anxiety, potentially mediated by the cognitive appraisal of self-efficacy, which subsequently influences social functioning ((school connectedness and behavioral adaptation (Internet addiction)), and mental and physical health (sleep problems), ultimately affecting adolescent anxiety levels. Based on this framework, we constructed a multiple mediation model to investigate these mechanisms from various perspectives, aiming to develop more targeted interventions for adolescents subjected to harsh parenting.

### The independent mediating role of self-efficacy

According to the cognitive appraisal theory of stress [16], the effects of stressors on an individual’s psychosocial adjustment are mediated by cognitive appraisal. Individuals who perceive themselves as incapable of managing stress are more prone to adverse psychological effects, including anxiety. Self-efficacy, rooted in Bandura’s social cognitive theory [17], refers to the belief in one’s competence to initiate, execute, persist, and achieve goals across various domains [18, 19]. This belief system, intrinsically linked to personal competence, human motivation, behavior, and achievement, plays a critical role in shaping responses to stress [17, 19].

Self-efficacy is often influenced by parenting styles [20]. Positive parenting, such as authoritative and warm involvement, is typically associated with enhanced self-efficacy and improved psychological adjustment [20–22]. In contrast, negative parenting, including parental rejection and neglect, constitutes a significant familial stressor that can undermine adolescents’ self-efficacy and psychosocial adjustment [21–23]. Adolescents who consistently encounter negative feedback, disapproval, and low expectations from parents may internalize feelings of unworthiness and incompetence, resulting in reduced self-efficacy [24–26]. Prior studies have also validated that harsh parenting undermines adolescents’ needs for independence from parental authority [27], impairs adaptive emotion regulation [28], and compromises coping strategies [29]. These negative experiences further diminish adolescents’ self-efficacy in emotion regulation [23], academic achievement [21], and social adaptation [28]. Therefore, harsh parenting may be linked to reduced self-efficacy in adolescents.

Moreover, higher self-efficacy is typically associated with lower anxiety levels. Individuals with high self-efficacy tend to maintain positive self-evaluations and exhibit greater confidence in their ability to manage challenges. Thus, high self-efficacy promotes psychological adaptation and reduces anxiety, whereas low self-efficacy correlates with heightened anxiety symptoms [30, 31]. Empirical studies have confirmed a significant negative correlation between self-efficacy and anxiety levels. In addition, research has shown that self-efficacy mediates the relationship between daily stressors and mental health outcomes [32], as well as between adverse childhood experiences and psychological distress [33]. Therefore, harsh parenting may lead to increased anxiety levels in adolescents by diminishing their self-efficacy. In this study, we hypothesize that self-efficacy mediates the relationship between harsh parenting and adolescent anxiety.

#### The independent mediating roles of school connectedness, Internet addiction, and sleep problems

The cognitive appraisal theory of stress posits that stressors can influence an individual’s coping strategies, such as problem-focused and emotion-focused responses, which affects social functioning and mental and physical health, ultimately impacting mental health [16]. This study will explore the mediating roles of school connectedness, Internet addiction, and sleep problems in the relationship between harsh parenting and adolescent anxiety.

Specifically, school connectedness can be considered as outcome of social adaptation to harsh parenting. School connectedness refers to students’ perception of acceptance, respect, inclusion, and support within the school environment [34]. Adolescents with high levels of school connectedness typically experience greater support from teachers and peers, fostering a sense of belonging [35]. However, those subjected to harsh parenting may perceive their parents as emotionally detached and critical, leading to reduced autonomy and respect, which undermines the parent-adolescent relationship [12, 36]. These feelings may extend to other social relationships, as research links harsh parenting to social anxiety and difficulties in peer relationships [12, 21, 28]. Moreover, adolescents subjected to harsh parenting or childhood trauma may replicate aggressive behaviors and engage in bullying or delinquency [37–39], further reducing their sense of attachment and belonging at school [21, 40]. A strong sense of school connectedness can serve as a buffer against both internalizing and externalizing problems [35, 41]. Poor relationships with peers and teachers, by contrast, may exacerbate anxiety [42]. Furthermore, previous studies have indicated that childhood abuse can influence adolescent depression through school connectedness [40]; however, few have investigated its mediating role between harsh parenting and adolescent anxiety. In this study, we hypothesize that school connectedness mediates the relationship between harsh parenting and adolescent anxiety.

Internet addiction can be viewed as behavioral adaptation to harsh parenting. Internet addiction is defined as excessive Internet use that leads to functional impairments in academic and psychosocial domains [43]. It manifests as compulsive use, uncontrollable urges to access the Internet, preoccupation with online activities, and conflicts in personal and professional issues [44]. Harsh parenting often disrupts the development of secure attachment within the family [12, 36], pushing adolescents to seek connections elsewhere. Moreover, harsh parenting impedes the cultivation of emotion regulation [45] and self-control [46], resulting in social anxiety and avoidance behaviors [21, 28]. Consequently, these adolescents may turn to the Internet to evade negative experiences and fulfill unmet psychological needs, potentially leading to Internet addiction [39, 47]. Research indicates that Internet-addicted adolescents generally experience more negative parenting styles, increased loneliness, and higher rates of bullying victimization [48]. As a maladaptive coping strategy, Internet addiction has been found to correlate with elevated anxiety symptoms [49, 50]. This may result from compulsive Internet use negatively impacting real-life relationships and fostering social comparison, which can trigger anxiety. Furthermore, studies have indicated that Internet addiction mediates the effect of childhood trauma on psychological distress [51]. However, the mediating role of Internet addiction between harsh parenting and anxiety symptoms remains underexplored. In this study, we hypothesize that Internet addiction mediates the relationship between harsh parenting and adolescent anxiety.

Sleep problems can be considered an outcome of mental and physical adaptation to the chronic stressor of harsh parenting [52–54]. Adolescents exposed to negative parenting often show heightened neural responses to mistakes [52] and suffer from physiological dysregulations due to chronic stress [4, 55], making them more susceptible to emotional problems [56]. These heightened cognitive and emotional reactions can contribute to sleep disturbances, either directly or indirectly [26, 57]. Disrupted sleep is commonly linked to higher levels of anxiety symptoms [53, 58]. Previous studies have indicated that sleep problems can alter brain network activity, including the limbic system and cognitive control networks (e.g., the dorsal anterior cingulate cortex and the anterior insula) as well as the regulation of neurotransmitter systems and emotional processing, which may heighten anxiety [59]. Furthermore, the mediating effect of sleep problems on the relationship between childhood adversity and adult stress has been established [60]. However, the potential mediating role of sleep problems between harsh parenting and adolescent anxiety remains unexplored. Thus, this study hypothesizes that sleep problems mediate the relationship between harsh parenting and adolescent anxiety.

### The sequential mediating roles

Self-efficacy plays a crucial role in human development [17] and may influence school connectedness, Internet addiction, and sleep quality. According to cognitive appraisal theory, individuals’ cognitive appraisal of stressors impacts their coping strategies. When adolescents perceive stressors as controllable and believe in their capability to handle challenges, they are more likely to adopt problem-focused strategies, such as fostering supportive connections at school to mitigate the negative effects of harsh parenting. Conversely, when they view stressors as uncontrollable, they may resort to emotion-focused coping, such as immersing themselves in the virtual world and compromising sleep quality.

Adolescents with high self-efficacy tend to believe in their capability to navigate difficulties, which enhances their academic performance and cultivates peer relationships. As a result, they are more likely to adapt well to school life, maintain positive perceptions of school [61], and display increased engagement [62], indicative of strong school connectedness. In contrast, those with low self-efficacy may struggle with real-life challenges, turning to the Internet for escapism, which heightens their risk of Internet addiction [63, 64]. Additionally, low self-efficacy and negative self-appraisal tend to reduce sleep quality [65]. Thus, harsh parenting may influence anxiety levels through a chain of mediating factors, including self-efficacy, school connectedness, Internet addiction, and sleep problems.

Taken together, while the impact of harsh parenting on adolescent anxiety is well-documented, few studies have explored their mechanisms by integrating the cognitive appraisal of self-efficacy, school connectedness, Internet addiction and sleep problems.Based on related theories and existing research, we propose a multiple mediation model with the following hypotheses: (1) Harsh parenting is associated with adolescent anxiety symptoms through the independent mediating roles of self-efficacy, school connectedness, Internet addiction, and sleep problems; and (2) harsh parenting is associated with adolescent anxiety symptoms through the chain mediating processes: self-efficacy → school connectedness, self-efficacy → Internet addiction, and self-efficacy → sleep problems.

### The effects of parental roles and living arrangements

Recent research indicates that mothers and fathers exhibit different influences on their children, even when using similar parenting styles [14, 28, 66]. Specifically, studies have identified differences in parenting styles and practices between mothers and fathers, with mothers tending to be more authoritative and fathers exhibiting more authoritarian behaviors [67]. In terms of the effects of parenting styles, research has found that paternal challenging parenting was linked to lower anxiety levels in children, whereas maternal challenging behaviors did not show such association [68]. Moreover, maternal harshness has been found to significantly correlate with increased depressive symptoms in children and adolescents; whereas paternal harshness did not demonstrate the same effect. Some research also found that paternal harshness exerted a greater effect on adolescents’ social anxiety compared to maternal harshness [28]. However, other studies have found that the impact of harsh parenting on anxiety outcomes does not significantly differ between mothers and fathers [14]. This underscores the need to further investigate the distinct effects of maternal and paternal harsh parenting on adolescent anxiety and the underlying mechanisms involved.

Furthermore, research has shown that living arrangements significantly affect adolescent mental health. Living arrangement is a broad concept that describes the patterns and types of cohabitation with family members [69]. It encompasses various dimensions, such as marital status (e.g., single, married, divorced), family type (e.g., single-parent), and household situation (e.g., living alone) [69]. Studies have found that adolescents in two-parent families generally report better subjective well-being [70] and lower levels of psychological maladjustment compared to those from other family structures [71]. In addition, helicopter parenting tends to have a greater impact on psychological adjustment for students living with their parents than for those living away [71]. Studies have also shown that the influence of father-child relationships [72] and father-adolescent conflicts [73] on mental health is stronger for adolescents living with their fathers. These findings suggest that the effects of parental behaviors on adolescents’ psychological and behavioral adaptation may vary depending on living arrangements. However, few studies have explored whether the effects of harsh parenting on adolescent anxiety differ across various living arrangements. Understanding these differences could inform targeted intervention strategies.

To further elucidate the potential effects of different parental roles and living arrangements on adolescent anxiety, we will compare (1) the effects of harsh fathering versus harsh mothering on adolescents’ anxiety; and (2) the effects of harsh parenting on adolescent anxiety in different living arrangements, including bi-parental parenting and single-parent parenting (living only with father or mother).

## Methods

### Participants

In November 2023, a psychological health survey was conducted on students of three middle schools in Qingzhen City, Guizhou Province. Considering the schools’ cooperation in the survey and research costs, we applied a convenience sampling method and selected three target schools. This survey used the entire group of sampling methods. All students from the first and second years of both junior and senior high school were invited to participate in the survey based on the voluntary principles. Students from the third year of junior and senior high school did not participate in this survey due to academic burden and exam pressure. A total of 3537 questionnaires were recovered by the online platform. After excluding incomplete and invalid responses (e.g., those with logical inconsistencies or patterned answering), we obtained 3,505 valid responses, yielding an efficiency of 99.10%. Both the students and their guardians signed an informed consent form, and this survey was approved by the Ethical Committee of the First People’s Hospital of Qingzhen City.

A total of 3505 adolescents completed this survey. Regarding their living arrangements, 2615 adolescents lived with both parents, 384 lived only with their mother (either biological mother or stepmother), 296 lived only with their father (either biological father or stepfather), and 210 lived with other people (e.g., grandparents or siblings). Given the focus of this study on the effects of parenting styles on anxiety symptoms in adolescents, participants living with both parents, only with mother, or only with father were included in the formal analyses (*N* = 3295, 54.7% girls), while those living with others were excluded. The ages in the final sample ranged from 11 to 19 (*M* = 14.97 years, *SD* = 1.37), with 10.1% in their first year of junior high school, 20.7% in their second year of junior high school, 53.1% in their first year of senior high school, and 16.1% in their second year of senior high school. Further details regarding the participants’ basic demographic characteristics are provided in Appendix Table S1.

### Measures

#### Harsh parenting

Following the example of prior research [11, 38], eight items were rated by participants to measure the levels of maternal harsh parenting (4 items) and paternal harsh parenting (4 items). Example items include, “When I did something wrong, my mother would lose her temper or even yell at me,” and “When I did something wrong, my father would hit me using his hands or feet.” Participants rated these items on a 5-point Likert scale (from 1 = *almost never did that* to 5 = *almost always did that*). Maternal and paternal scores on the subscales were calculated separately, with higher scores indicating more severe levels of maternal or paternal harsh parenting. This scale has been demonstrated to have satisfactory validity and reliability in prior studies with Chinese adolescents [11, 38, 45]. In this study, among adolescents who lived with both parents, the internal consistency coefficients for maternal and paternal harsh parenting were 0.839 and 0.869, respectively. For adolescents who lived only with their mothers, the internal consistency coefficients for maternal and paternal harsh parenting were 0.866 and 0.915, respectively.For adolescents who lived only with their fathers, the internal consistency coefficients for maternal and paternal harsh parenting were 0.871 and 0.847, respectively.

#### General self-efficacy

The General Self-efficacy Scale [18], a unidimensional scale, was used to measure the general levels of individuals’ belief in their ability to address life demands and achieve desired attainments. The scale comprises 10 items; a sample item is, “It is easy for me to stick to my aims and accomplish my goals.” Participants rated each item on a 4-point Likert scale, from 1 = *not at all true* to 4 = *exactly true*. Higher scores indicate greater levels of self-efficacy. This scale has been found to have good validity and reliability in prior studies [18, 31]. In this study, the internal consistency coefficient of this scale for the total sample was 0.939.

#### School connectedness

The School Connectedness Scale [35] was used to assess adolescents’ levels of school connectedness. The scale comprises 10 items, measuring the subscales of teacher support (3 items), peer support (4 items), and sense of belonging to school (3 items). A sample item was, “I am proud to be part of the school”. All items were assessed on a 5-point Likert scale from 1 = *strongly disagree* to 5 = *strongly agree*, with higher scores indicating higher levels of school connectedness. This scale has been found to have satisfactory validity and reliability [35, 40]. In this study, the internal consistency coefficient of the scale for the total sample was 0.889.

#### Internet addiction

The Chinese version [74] of the Internet Addiction Scale [43] was used to assess the levels of Internet addiction. The scale comprises eight items (e.g., “Feel the need to use the Internet with increasing amounts of time in order to achieve satisfaction”). All items were rated on a 6-point Likert scale from 1 = *completely disagree* to 6 = *completely agree*, with higher scores indicating more severe symptoms of compulsive Internet use. This measure has been demonstrated to have high reliability and validity [43, 74]. In this study, the internal consistency coefficient of the scale for the total sample was 0.916.

#### Sleep problems

The Self-Rating Scale of Sleep [75] was used to assess the sleep quality of adolescents over the past month. The scale consisted of 10 questions; a sample item is, “Do you have difficulty falling asleep in the past month?” All items were scored on a 5-point scale (1 ~ 5), with total scores ranging from 10 to 50. Higher scores indicate more severe sleep problems. This scale has established the norm for a group of Chinese participants and has been shown with good reliability and validity [75]. In this study, the internal consistency coefficient of this scale for the total sample was 0.818.

#### Anxiety symptoms

The Generalized Anxiety Disorder-7 scale [76] was used to assess adolescents’ symptoms of anxiety. Participants were asked to report how often they experienced anxiety symptoms in the past two weeks. It comprises seven items; a sample item is, “Over the last two weeks, how often have you been bothered by feeling nervous, anxious, or on edge”. All items were scored on a 4-point scale from 0 = *not at all* to 3 = *nearly every day*. Higher scores indicate more severe anxiety symptoms. This scale has been widely used and found to have strong validity and reliability [50, 76]. In this study, the internal consistency coefficient of the scale for the total sample was 0.940.

#### Living arrangements

Living arrangements were assessed by asking the adolescents to report “Who do you live with?”. Adolescents who answered that they lived with both parents were categorized as “living with parents” group. Those who answered that they lived only with mothers (biological mother or stepmother) were categorized as “living only with mother” group. Those who answered that they lived only with fathers (biological father or stepfather) were categorized as “living only with father” group.

##### Analysis plan

First, we conducted a preliminary analysis of the entire sample, which included univariate regression analyses of demographic variables on anxiety symptoms, and Pearson’s bivariate correlation analysis among the core variables using SPSS 23.0. Next, we constructed a multiple mediation model based on latent variables and employed structural equation modeling (SEM) analyses to evaluate the goodness of fit for the mediation model. Common indicators for assessing model fit included the normed chi-square (*χ*²), degrees of freedom (*df*), comparative fit index (CFI), Tucker-Lewis index (TLI), root mean square error of approximation (RMSEA), and standardized root mean square residual (SRMR). Subsequently, we conducted a multigroup analysis based on participants’ living arrangements (living with both parents, living only with the mother, and living only with the father), and further performed multiple mediation tests within each group. The significance and effect sizes of each mediation path were examined using the bias-corrected bootstrap method with 5000 resamples.

## Results

### Common method bias test

The Harman single-factor test showed that after conducting a principal component analysis on all items, a total of nine factors with eigenvalues greater than 1 were extracted, explaining 65.5% of the variance. The first extracted common factor accounted for 29.1% of the variance (below the 40.0% threshold), indicating no serious issue of common method bias in the present study.

### Preliminary analyses

First, univariate regression analyses were performed to predict anxiety based on demographic variables across the entire sample (see Appendix Table S1). The results showed that gender, grade, age, and mother and father working outside time per year significantly predicted anxiety and were therefore included as controlling variables in the subsequent analyses. Correlation analyses (see Table 1) showed that both maternal and paternal harsh parenting were positively correlated with anxiety symptoms. Moreover, significant intercorrelations were observed between maternal and paternal harsh parenting, self-efficacy, school connectedness, Internet addiction, sleep problems, and anxiety symptoms (all *p* values < 0.001).

**Table 1:** Means, standard deviations, and Pearson bivariate correlation coefficients for the study variables in the entire sample

Variables	1	2	3	4	5	6	7
1. Maternal HP	—						
2. Paternal HP	0.67^***^	—					
3. Self-efficacy	−0.23^***^	−0.20^***^	—				
4. School connectedness	−0.30^***^	−0.28^***^	0.53^***^	—			
5. Internet addiction	0.36^***^	0.32^***^	−0.30^***^	−0.40^***^	—		
6. Sleep problems	0.32^***^	0.28^***^	−0.31^***^	−0.46^***^	0.40^***^	—	
7. Anxiety symptoms	0.42^***^	0.40^***^	−0.37^***^	−0.52^***^	0.48^***^	0.56^***^	—
Mean	1.52	1.43	2.68	3.94	2.31	2.05	0.56
Standard Deviation	0.67	0.68	0.69	0.71	1.12	0.61	0.64

Note. *N* = 3295. HP = harsh parenting. ^***^p < 0.001

### Testing the mediation model in the entire sample

**Fig. 1:**
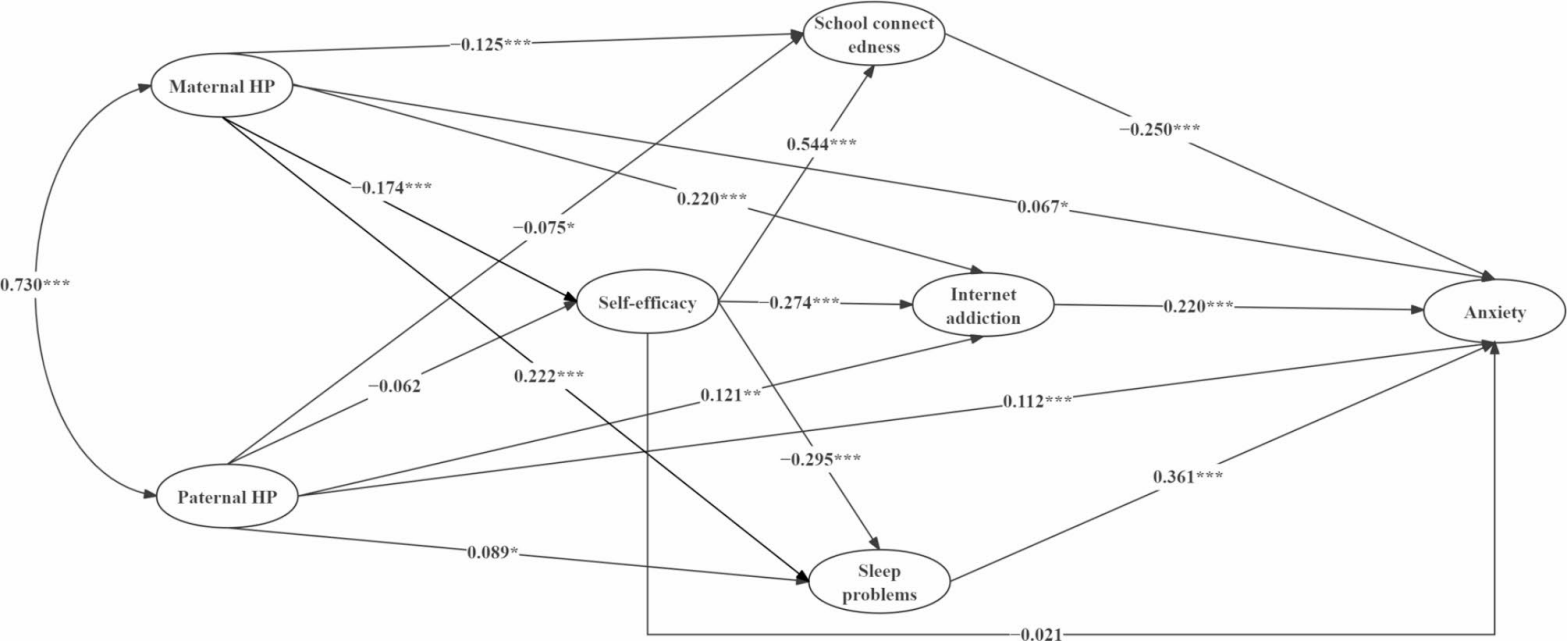
Structural equation modeling diagram for adolescents in the entire sample. The observed variables for maternal and paternal harsh parenting were derived directly from their original items. The three dimensions of school connectedness (i.e., teacher support, peer support, and school belongingness) served as observed variables. For the remaining constructs, three observed variables were created using the factor balancing method based on the factor loadings of each item. HP = harsh parenting. Standardized regression coefficients were reported. ^*^p < 0.05, ^**^p < 0.01, ^***^p < 0.001

A SEM mediation model based on the latent variables was constructed in the entire sample (*N *= 3295). The goodness-of-fit assessment for the measurement models for the latent variables showed that all factor loadings exceeded 0.6 (*p* < 0.001) with satisfactory model fit indices: *χ*² = 3190.298, *df* = 209, *p* < 0.001, CFI = 0.949, TLI = 0.938, RMSEA = 0.066, and SRMR = 0.046. In addition, tests for measurement model invariance—configural, metric, and scalar—based on three different living arrangements demonstrated cross-group invariance of the factor structure (ΔCFI ≤ 0.015, ΔTLI ≤ 0.015, ΔRMSEA ≤ 0.010). Furthermore, the structural model in the multiple mediation analyses exhibited good fit indices: *χ*² = 4781.656, *df* = 322, *p* < 0.001, CFI = 0.925, TLI = 0.914, RMSEA = 0.065, and SRMR = 0.070. As depicted in Fig. 1, all path coefficients in the model were significant, except for the direct effects of paternal harsh parenting on self-efficacy and self-efficacy on anxiety, which were not significant.

### The moderating role of living arrangements

We performed a multigroup analysis to compare the mediation mechanisms through which harsh parenting affects adolescent anxiety across the three living arrangements. Specifically, we tested for differences in chi-square values between a freely estimated model (without constraining any parameters across groups) and a constrained model (setting the regression coefficients to be identical across groups) (∆*χ*² = 50.076, ∆*df* = 34, *p* < 0.05), indicating significant moderating effects.

We subsequently analyzed specific paths with moderating effects. The results showed that the predictive effect of paternal harsh parenting on self-efficacy differed between those living with both parents and those living with their father (β = 0.192, *p* < 0.05), as well as between those living with both parents and those living with their mother (β = 0.197, *p* < 0.05). Additionally, the predictive effect of paternal harsh parenting on sleep problems differed between those living with both parents and those living with their father (β = −0.183, *p* < 0.05), while maternal harsh parenting’s effect on sleep problems varied between those living with both parents and those living with their father (β = 0.205, *p* < 0.05).

**Fig. 2:**
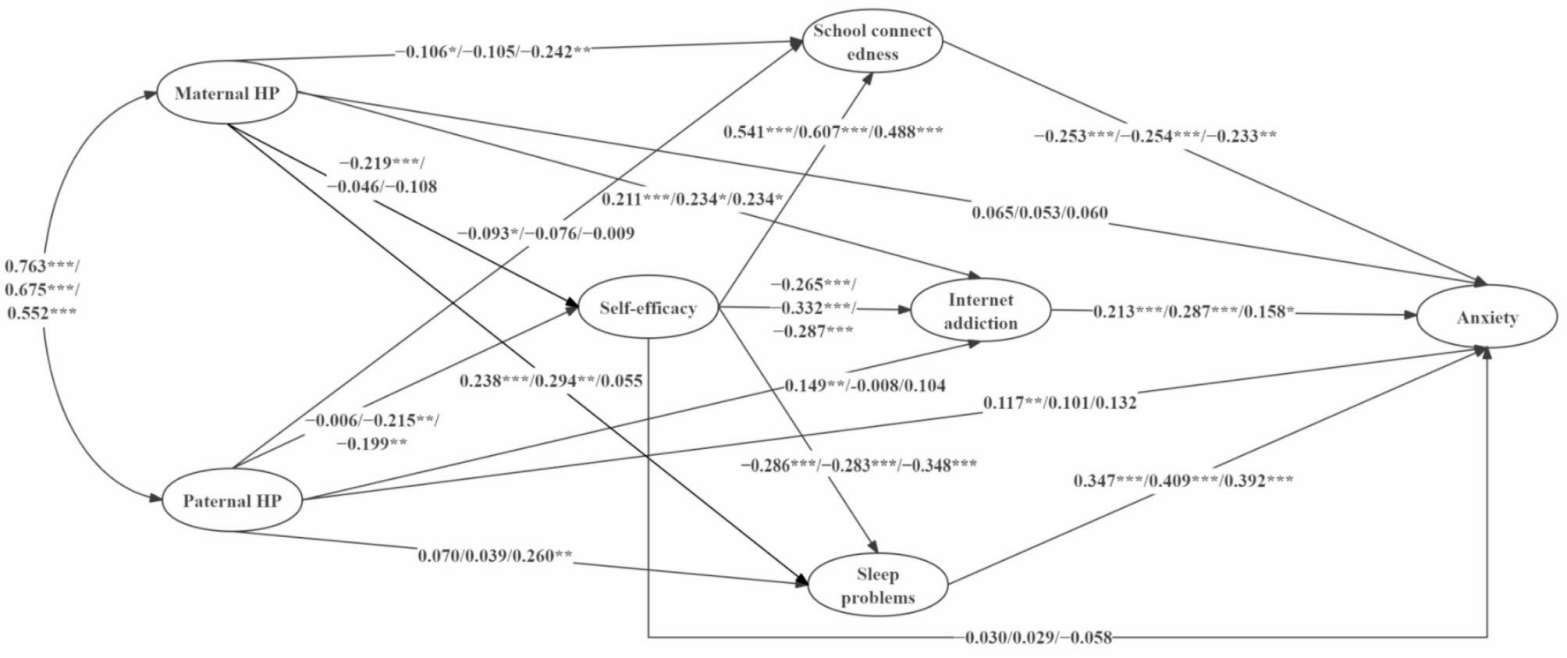
Structural equation modeling diagram for adolescents across three different living arrangements. Standardized regression coefficients indicated on each path in the figure were derived from the groups living with both parents, only with the mother, and only with the father, respectively. HP = harsh parenting. ^*^p < 0.05, ^**^p < 0.01, ^***^p < 0.001

Thus, this study further examined the specific mediating effects and pathways through which harsh parenting influences adolescent anxiety under these different living arrangements. The multiple mediation model showed good fit indicators across the three living arrangements: *χ*^2^ = 4175.861, *df* = 322, *p* < 0.001, CFI = 0.918, TLI = 0.906, RMSEA = 0.068, and SRMR = 0.074 (living with both parents group); *χ*^2^ = 766.321 *df* = 322, *p* < 0.001, CFI = 0.941, TLI = 0.933, RMSEA = 0.060, and SRMR = 0.074 (living only with mother group); and *χ*^2^ = 806.006, *df* = 322, *p* < 0.001, CFI = 0.906, TLI = 0.893, RMSEA = 0.071, and SRMR = 0.079 (living only with father group). The path coefficients are displayed in Fig. 2 (see Appendix Table S2 for more details).

In the living with both parents group, as shown in Table 2, the indirect effect analyses showed that maternal harsh parenting predicted anxiety symptoms through the independent mediating roles of school connectedness, Internet addiction, and sleep problems, as well as the sequential mediating roles of self-efficacy → school connectedness, self-efficacy → Internet addiction, and self-efficacy → sleep problems, which accounted for 9.3%, 15.5%, 28.3%, 10.3%, 4.1%, and 7.6% of the total effect of maternal harsh parenting on anxiety symptoms, respectively. The direct effect became non-significant in the presence of these mediators. Moreover, regarding the effect of paternal harsh parenting on anxiety symptoms, the independent mediating roles of school connectedness and Internet addiction accounted for 11.6% and 16.1% of the total effect of paternal harsh parenting on anxiety, respectively. However, the indirect effect through other pathways was not significant. The direct effect remained significant in the presence of these mediators.

**Table 2:** The direct and indirect effects between harsh parenting and adolescent anxiety across three living arrangements

Pathway	Adolescents who lived with their both parents (*N* = 2615)			Adolescents who lived only with their mother (*N* = 384)			Adolescents who lived only with their father (*N* = 296)	
	Effect (standardized)	95% CI		Effect (standardized)	95% CI		Effect (standardized)	95% CI
**Total effect**:								
Maternal HP → Anxiety	0.290	[0.188, 0.398]		0.282	[0.056, 0.470]		0.213	[− 0.017, 0.438]
Paternal HP → Anxiety	0.199	[0.090, 0.303]		0.206	[0.036, 0.407]		0.322	[0.122, 0.523]
**Direct effect**:								
Maternal HP → Anxiety	0.065	[− 0.008, 0.137]		0.053	[− 0.084, 0.188]		0.060	[− 0.131, 0.242]
Paternal HP → Anxiety	0.117	[0.048, 0.187]		0.101	[− 0.013, 0.231]		0.132	[− 0.028, 0.306]
**Indirect effect**:								
Total indirect effect：Maternal HP → Anxiety	0.225	[0.156, 0.299]		0.229	[0.069, 0.379]		0.153	[0.022, 0.294]
Total indirect effect: Paternal HP → Anxiety	0.081	[0.011, 0.148]		0.105	[− 0.039, 0.237]		0.190	[0.062, 0.341]
Maternal HP → Self-efficacy → Anxiety	0.006	[− 0.004, 0.019]		−0.001	[− 0.024, 0.005]		0.006	[− 0.006, 0.040]
Maternal HP → School connectedness → Anxiety	0.027	[0.006, 0.053]		0.027	[− 0.006, 0.081]		0.056	[0.019, 0.129]
Maternal HP → Internet addiction → Anxiety	0.045	[0.027, 0.070]		0.067	[0.006, 0.155]		0.037	[0.006, 0.105]
Maternal HP → Sleep problems → Anxiety	0.082	[0.047, 0.125]		0.121	[0.031, 0.212]		0.022	[− 0.035, 0.088]
Maternal HP → Self-efficacy → School connectedness → Anxiety	0.030	[0.018, 0.046]		0.007	[− 0.016, 0.040]		0.012	[− 0.002, 0.042]
Maternal HP → Self-efficacy → Internet addiction → Anxiety	0.012	[0.007, 0.019]		0.004	[− 0.010, 0.023]		0.005	[0.000, 0.022]
Maternal HP → Self-efficacy → Sleep problems → Anxiety	0.022	[0.013, 0.033]		0.005	[− 0.012, 0.028]		0.015	[− 0.002, 0.042]
Paternal HP → Self-efficacy → Anxiety	0.000	[− 0.003, 0.005]		−0.006	[− 0.040, 0.014]		0.012	[− 0.011, 0.052]
Paternal HP → School connectedness → Anxiety	0.023	[0.002, 0.048]		0.019	[− 0.015, 0.073]		0.002	[− 0.038, 0.050]
Paternal HP → Internet addiction → Anxiety	0.032	[0.013, 0.052]		−0.002	[− 0.062, 0.051]		0.016	[− 0.005, 0.064]
Paternal HP → Sleep problems → Anxiety	0.024	[− 0.008, 0.057]		0.016	[− 0.057, 0.096]		0.102	[0.034, 0.201]
Paternal HP → Self-efficacy → School connectedness → Anxiety	0.001	[− 0.012, 0.012]		0.033	[0.012, 0.071]		0.023	[0.005, 0.062]
Paternal HP → Self-efficacy → Internet addiction→ Anxiety	0.000	[− 0.005, 0.005]		0.020	[0.007, 0.046]		0.009	[0.002, 0.030]
Paternal HP → Self-efficacy → Sleep problems → Anxiety	0.001	[− 0.008, 0.009]		0.025	[0.009, 0.053]		0.027	[0.008, 0.063]

Note. Standardized regression coefficients were reported; Bootstrap sample size = 5000; HP = harsh parenting; CI = Confident interval

In the living only with mother group, as shown in Table 2, maternal harsh parenting predicted anxiety symptoms through the independent mediating roles of Internet addiction and sleep problems, which accounted for 23.8% and 42.9% of the total effects of maternal harsh parenting on anxiety symptoms, respectively. Moreover, regarding the effect of paternal harsh parenting on anxiety symptoms, the sequential mediating roles of self-efficacy → school connectedness, self-efficacy → Internet addiction, and self-efficacy → sleep problems accounted for 16.0%, 9.7%, and 12.1% of the total effects of paternal harsh parenting on anxiety symptoms, respectively. However, the indirect effects via other pathways were not significant. The direct effect of both maternal and paternal harsh parenting on anxiety symptoms became non-significant in the presence of mediators.

In the living only with father group, as shown in Table 2, maternal harsh parenting predicted anxiety symptoms through the independent mediating roles of school connectedness and Internet addiction, which accounted for 26.3% and 17.4% of the total effects of maternal harsh parenting on anxiety symptoms, respectively. Moreover, regarding the effect of paternal harsh parenting on anxiety symptoms, the independent mediating roles of sleep problems and the sequential mediating roles of self-efficacy → school connectedness, self-efficacy → Internet addiction, and self-efficacy → sleep problems accounted for 31.7%, 7.1%, 2.8%, and 8.4% of the total effect of paternal harsh parenting on anxiety symptoms, respectively. However, the indirect effect through other pathways was not significant. The direct effects of both maternal and paternal harsh parenting on anxiety symptoms became non-significant in the presence of mediators.

## Discussion

The present study constructed a multiple mediation model to examine the internal mechanisms through which harsh parenting affects adolescent anxiety symptoms. The findings extend the prior research by investigating the distinct parental roles of harsh mothering and harsh fathering in adolescents’ anxiety symptoms and by distinguishing among different living arrangements. The results elucidate the internal processes underlying the relationships between harsh parenting and adolescent anxiety symptoms and identify the effects of living arrangements. The findings have important implications for interventions targeting anxiety symptoms in adolescents exposed to harsh parenting.

### Harsh parenting and anxiety symptoms

The present study revealed that both maternal and paternal harsh parenting were associated with increased anxiety symptoms in adolescents, which supported the parental acceptance–rejection theory [6]. The findings align with previous research, which has consistently identified harsh parenting as a significant risk factor for anxiety and depressive symptoms in adolescents across different cultural and regional contexts [13, 14, 56, 77]. The results also demonstrated the crucial roles of both maternal and paternal parenting in adolescents’ mental health [14]. However, differences were observed in the specific relationships between maternal versus paternal harsh parenting and adolescent anxiety in the multiple mediation model. Specifically, in the living with both parents group, paternal harsh parenting predicted anxiety levels both directly and indirectly, indicating partial mediation effects. However, the direct effects of paternal harsh parenting in the living only father and only mother groups and maternal harsh parenting in the three living arrangements on anxiety levels were non-significant. These results validate the influence of different family structures (e.g., single-parent) and living arrangements on adolescent psychological and behavioral development [41, 72, 78]. These findings suggest that when harsh parenting is delivered by both parents, harsh parenting by mothers versus fathers affects adolescent anxiety through distinct mechanisms [29, 37, 45]. When either the mother or father assumes the primary caregiving role alone, the direct effects of harsh parenting on adolescent anxiety are diminished, operating more through mediating pathways. The findings also confirm that maternal and paternal parenting can have more profound effects on mental health among adolescents who live in two-parent families than those living with one parent [70].

### Independent mediating effects

Consistent with our hypotheses and the cognitive appraisal theory of stress [16], the mediation analysis partially confirmed that school connectedness, Internet addiction, and sleep problems serve as social, mental, and physical mechanisms mediating the relationship between harsh parenting and adolescent anxiety across all three living arrangements.

Specifically, this study found that maternal harsh parenting (in the living with both parents group and living only with father group) and paternal harsh parenting (in the living with both parents group) can affect adolescents’ anxiety symptoms by decreasing their sense of school connectedness. This finding aligns with prior studies indicating that school connectedness is an important mechanism through which adverse family environments (e.g., disadvantaged family structures, childhood abuse) influence adolescents’ mental health problems [40, 41]. Chronic exposure to corporal punishment and verbal abuse from parents may foster insecure parent-child attachment [12], which, in turn, affects adolescents’ peer relationships [21]. These adolescents may also internalize their parents’ rough attitudes and behaviors, leading to engagement in aggressive or delinquent behaviors at school [79]. Thus, maladaptive parenting practices impede adolescents’ classroom engagement [11] and undermine their sense of connectedness to school [40]. When adolescents perceive a lack of respect, acceptance, and support from teachers and peers at school, they become more susceptible to mental health problems [41].

However, in the group living with the mother, school connectedness did not mediate the relationship between either maternal or paternal harsh parenting and anxiety symptoms. Similarly, in the group living with the father, school connectedness did not mediate the relationship between paternal harsh parenting and anxiety symptoms. According to attachment theory [80], adolescents typically develop a stronger bond with their mothers, who are often perceived as more accepting, responsive, and supportive compared to fathers [67]. Consequently, when living only with their mothers, adolescents may develop distinct psychological adaptations and coping mechanisms to navigate negative maternal behaviors. Especially in the context of Chinese culture, the doctrine of Confucian filial piety emphasizes respect and obedience toward parents [81], which may mitigate the impact of maternal harsh parenting on their social adjustment. In contrast, paternal harshness is more prevalent, with fathers generally exhibiting more restrictive, coercive, and punitive behaviors, while demonstrating less parental concern than mothers [67]. Thus, paternal harsh discipline is commonly regarded as normative and even indicative of parental care and concern [82], which may mitigate its adverse effects on adolescents’ social adaptation in living only with the mother or the father group.

This study also confirmed the mediating role of Internet addiction in the association between maternal harsh parenting (in the living with both parents group, living only with the mother, living only with father group) and paternal harsh parenting (in the living with both parents group) and adolescents’ anxiety symptoms. Adolescents raised by harsh parents are subjected to emotional and physical hostility, rejection, and neglect in their real-life relationships. Such experiences frustrate their basic psychological needs [47] and disrupt their emotional regulation [45, 83] and self-control abilities [46]. Consequently, these adolescents may seek refuge in the virtual realm of the Internet, potentially resulting in Internet-related disorders [47, 83, 84]. This heightened propensity for Internet addiction can subsequently impair adolescents’ academic performance and interpersonal relationships, and ultimately contribute to elevated anxiety symptoms [50].

However, in groups living only with their mother or father, the mediating effect of Internet addiction between paternal harsh parenting and anxiety was non-significant. This aligns with the notion that adolescents who live only with one parent may view paternal harshness as normative experiences and have adapted to them, thereby diminishing its impact as a stressor on their behavioral adaptation. In contrast, mothers’ negative practices like punishment, aggression, blame, and neglect may be viewed as less normative and more threatening to adolescents’ development [26, 85]. Adolescents may expect more emotional support from their mothers, resulting in conflict when confronted with maternal harshness. They may escape into the virtual world as a coping mechanism to avoid negative experiences related to maternal parenting.

The present study also found that maternal harsh parenting (in those living with both parents and only with mother) and paternal harsh parenting (in those living only with father) can intensify adolescents’ anxiety symptoms by decreasing their sleep quality. The results are in line with previous research that has identified sleep problems as pathways through which negative family environments (e.g., childhood adversity) influence mental health outcomes in adolescents [53, 60, 86]. When adolescents perceive insecurity and threats from their external environment, they may become highly vigilant and aroused by potentially threatening stimuli, resulting in increased sleep disturbances [87], particularly within the family context [26, 88]. Given the critical role of sleep in individual development, sleep problems have a wide-ranging impact on adolescents’ cognition, emotions, and behaviors [87, 89]. Thus, adolescents with more sleep problems are at a higher risk of developing both internalizing and externalizing problems [53, 87].

However, in families where adolescents live with both parents or only their mother, sleep problems did not significantly mediate the relationship between paternal harsh parenting and anxiety. Similarly, in families with only the father present, sleep problems did not mediate the relationship between maternal harsh parenting and anxiety. This suggests that sleep problems are more susceptible to the influence of the primary caregiver. According to the traditional rearing model and social roles, mothers typically assume more responsibilities as caregivers, hence cultivating a more profound connection with their children [15, 38]. Thus, in families where both parents are present or only mothers are present, mothers often take on more caregiving responsibilities and exhibit increased engagement [90], making maternal aggression and negativity more likely to lead to sleep problems and anxiety in adolescents. In contrast, in families where adolescents live only with their fathers, increased paternal involvement in caregiving leads to more interaction. As a result, the father’s role in restrictive and harsh parenting becomes more prominent [67], making adolescents more susceptible to sleep problems related to harsh parenting.

Moreover, this study found that the independent mediating effect of self-efficacy on the link between harsh parenting and anxiety was non-significant across all three living arrangements. Both maternal harsh parenting (in those living with both parents) and paternal harsh parenting (in those living only with father and only with mother) were found to negatively predict self-efficacy. This is consistent with prior studies demonstrating that positive parenting enhances adolescents’ self-efficacy, while negative parenting diminishes it [24, 25, 91]. Contrary to our hypothesis, the predictive effect of self-efficacy on anxiety was non-significant across all three living arrangements. This coincides with some prior studies suggesting that self-efficacy may influence mental health problems through indirect processes, such as risk perception and coping strategies [31] or psychological inflexibility [92], with the direct effect of self-efficacy being non-significant.

### Sequential mediating effects

The present study also identified the sequential mediating pathways that link harsh parenting to adolescent anxiety, partially supporting our hypothesis. The results show that maternal harsh parenting (in those living with both parents) and paternal harsh parenting (in those living only with father and only with mother) can affect self-efficacy, which in turn affects school connectedness, Internet addiction, and sleep problems, ultimately contributing to heightened anxiety symptoms. Adolescents who experience chronic harsh parenting often exhibit reduced self-acceptance [93] and harbor negative perceptions regarding their problem-solving abilities. This negative self-evaluation further impairs their biopsychosocial adjustments, resulting in mental health problems. The findings support the postulate of the social cognitive theory that self-efficacy is essential to human functioning and has a wide-ranging impact on individuals’ psychosocial adaptation by influencing their cognition, motivation, and emotion [17]. The sequential mediating effects also highlighted the important roles of cognitive appraisal and stress coping mechanisms in the effect of harsh parenting on anxiety.

Studies have shown that adolescents with high self-efficacy tend to possess higher levels of self-esteem [94], adopt more positive cognitive-emotional coping strategies [95], and experience enhanced well-being with school and peers [94]. Thus, when subjected to harsh parenting, adolescents with high self-efficacy may compensate for their challenging family environment by fostering a sense of belonging and connection with their school (i.e., school connectedness). Conversely, adolescents with low self-efficacy generally exhibit diminished self-esteem and are more inclined to resort to negative cognitive-emotional coping strategies [96]. For example, they may attempt to evade their problems by immersing themselves in virtual networks, potentially resulting in Internet addiction [97], and experiencing disruptions in their sleep quality [65]. These maladaptive coping mechanisms can further lead to mental health problems (e.g., anxiety). These findings indicate that, although self-efficacy did not directly influence adolescent anxiety symptoms, it could indirectly influence anxiety symptoms through mediators such as school adjustment, mental and physical health, thus lending support to the multiple mediation model.

However, in families where adolescents live with both parents, the serial mediating effects between paternal harsh parenting and adolescent anxiety were not significant. Similarly, this effect regarding maternal harsh parenting was not observed in families where adolescents live only with their mothers or fathers. According to attachment theory [80] and the traditional rearing model [15, 38], mothers typically engage more in daily care and nurturing activities [90], which exerts a more pronounced effect on adolescent mental health when they live in intact families with both parents fulfilling their duties [9, 12, 15, 98]. Thus, in two-parent households, when adolescents consistently perceive hostility, aggression, and denial from their primary caregivers (usually mothers), the self-assessment of their capabilities of coping with challenges diminishes. Under such conditions, paternal harsh parenting may be mitigated by maternal support or other social factors, reducing its negative effects on self-evaluation and mental health. In contrast, in families where adolescents live only with one parent, the social role of the father is differentiated and emphasized. According to the rearing model and gender role expectations, fathers primarily assume the authoritative role, enforcing rules through physical and verbal discipline [15, 38]. This increased involvement in discipline and training significantly shapes adolescents’ development and behavior [37]. Thus, harsh parenting from fathers can lead to feelings of helplessness or decreased self-confidence in adolescents, ultimately lowering their self-efficacy.

### Limitations and future directions

This study has some limitations that should be noted. First, due to the cross-sectional design of this study, the findings cannot rigorously determine the causal relationships between variables. Future research should incorporate longitudinal studies to validate the constructed models. Second, all data in this study were collected from adolescents’ self-reports, which are susceptible to bias. Future research should use additional assessments from other sources. For example, when measuring harsh parenting levels, integrating reports from parents [38], spouses [28], and adolescents [11] could more comprehensively reveal harsh parenting. Third, since our study sample consists of school students, the generalizability of the research findings is limited to school settings. Further investigation involving clinical populations is needed to confirm the applicability of the multiple mediation model in clinical practice. Fourth, this study focused on exploring how harsh parenting affects anxiety through psychological dimensions while neglecting the potential biological mechanisms such as dysregulation of the hypothalamic-pituitary-adrenal (HPA) axis and epigenetic factors. Moreover, this study focused solely on the independent mediating effects of school connectedness, Internet addiction, and sleep problems, without examining the interrelationships among these variables. Future research should explore additional potential mediators and examine how these factors interact in the context of parenting styles and adolescent mental health. Finally, this study only assessed adolescents’ current living arrangements without addressing the specific reasons for these arrangements. Future studies could conduct a more comprehensive evaluation of the family structure and living arrangements of participants to further investigate the impact of harsh parenting on adolescent mental health across different family dynamics.

#### Implications

The findings have important implications. For example, it is imperative to implement parenting interventions that improve parenting attitudes, promote parent-child interactions, and diminish harsh parenting practices [99]. Moreover, given the key mediating processes identified in this study, community workers, clinical practitioners, teachers, and parents should pay more attention to facilitating adolescents’ self-development and biopsychosocial adaptation. Specifically, intervention strategies include cultivating adolescents’ generalized self-efficacy and perceived competence across academic, social, and emotional regulation domains. Encouraging adolescents to strengthen their bonds with peers, teachers, and school can enhance their sense of school connectedness. Moreover, to alleviate Internet addiction, adolescents can cultivate adaptive emotion regulation strategies and enhance self-control abilities. Efforts from families and communities should focus on optimizing the sleep environment for adolescents and improving adolescents’ cognitive and emotional regulation skills, thereby improving their sleep quality, and reducing anxiety symptoms. Furthermore, more targeted intervention programs should be designed to consider the unique parenting roles of mothers and fathers, as well as the distinct living arrangements. These programs should recognize the influences of maternal and paternal parenting on their children’s development and address the specific needs of adolescents living in different family arrangements, which aims at enhancing mental health in adolescents.

## Conclusions

In conclusion, this study constructed a multiple mediation model to examine the internal mechanisms through which harsh parenting affects adolescent anxiety symptoms. Its findings add to previous studies by investigating the distinct roles of maternal and paternal parenting in adolescents’ anxiety symptoms and differentiating the living arrangements of adolescents. In addition, this study tested the multiple mediation model to elucidate the independent mediating roles of school connectedness, Internet addiction, and sleep problems, as well as the sequential mediating pathways involving self-efficacy → school connectedness, self-efficacy → Internet addiction, and self-efficacy → sleep problems in the association between harsh parenting and adolescent anxiety. The findings have important practical implications for the development of prevention and intervention strategies targeting adolescent anxiety.

## Electronic supplementary material

Below is the link to the electronic supplementary material.

Supplementary Material 1

## Data Availability

No datasets were generated or analysed during the current study.
