# Supplementary material for: Maternal and paternal harsh parenting and anxiety symptoms in Chinese adolescents: examining a multiple mediation model
Source: Child Adolesc Psychiatry Ment Health. 2024 Oct 22;18:134. doi: 10.1186/s13034-024-00826-9 (PMC11515719; doi:10.1186/s13034-024-00826-9)
Supplement: Supplementary file 1 — Supplementary Material 1 [file 13034_2024_826_MOESM1_ESM.doc]

**Table S1** Preliminary demographic characteristics of the participants and univariate regression analysis of factors predicting anxiety (*N* = 3295).

|  | Frequency (Percentage) | Mean | Standard Deviation | β | *t* |
| --- | --- | --- | --- | --- | --- |
| **Age (year)** |  | 14.79 | 1.37 | 0.052 | 3.002^**^ |
| **Gender** |  |  |  | 0.089 | 5.124^***^ |
| Boys | 1491 (45.3%) |  |  |  |  |
| Girls | 1804 (54.7%) |  |  |  |  |
| **Grade** |  |  |  | 0.049 | 2.808^**^ |
| First-year junior high school | 333 (10.1%) |  |  |  |  |
| Second-year junior high school | 682 (20.7%) |  |  |  |  |
| First-year senior high school | 1751 (53.1%) |  |  |  |  |
| Second-year senior high school | 529 (16.1%) |  |  |  |  |
| **Only-child status** |  |  |  | 0.002 | 0.101 |
| Yes | 443 (13.4%） |  |  |  |  |
| No | 2852 (86.6%) |  |  |  |  |
| **Dormitory residence status** |  |  |  | 0.005 | 0.276 |
| Yes | 2643 (80.2%) |  |  |  |  |
| No | 652 (19.8%) |  |  |  |  |
| **Maternal education level** |  |  |  | −0.020 | −1.171 |
| Uneducated | 64(1.9%) |  |  |  |  |
| Primary school and below | 858 (26.0%) |  |  |  |  |
| Junior high school | 1546 (46.9%) |  |  |  |  |
| Senior high school | 537 (16.3%) |  |  |  |  |
| College | 279 (8.5%) |  |  |  |  |
| Master’s degree and above | 11 (0.3%) |  |  |  |  |
| **Paternal education level** |  |  |  | −0.017 | −0.954 |
| Uneducated | 22(0.7%) |  |  |  |  |
| Primary school | 690 (20.9%) |  |  |  |  |
| Junior high school | 1650 (50.1%) |  |  |  |  |
| Senior high school | 631 (19.2%) |  |  |  |  |
| College | 290 (8.8%) |  |  |  |  |
| Master’s degree and above | 12 (0.4%) |  |  |  |  |
| **Mother working outside the home on average per year (months)** |  |  |  | 0.071 | 4.084^***^ |
| Never | 2167 (65.8 %) |  |  |  |  |
| < 2 months | 405 (12.3%) |  |  |  |  |
| 2~4 months | 165 (5.0%) |  |  |  |  |
| 4~6 months | 161 (4.9%) |  |  |  |  |
| > 6 months | 397 (12.0%) |  |  |  |  |
| **Father** **working outside the home on average per year (months)** |  |  |  | 0.080 | 4.606^***^ |
| Never | 1580 (48.0%) |  |  |  |  |
| < 2 months | 568 (17.2%) |  |  |  |  |
| 2~4 months | 277 (8.4%) |  |  |  |  |
| 4~6 months | 259 (7.9%) |  |  |  |  |
| > 6 months | 611 (18.5%) |  |  |  |  |

*Note*. Gender (0 = *boys* and 1 = *girls*), only-child status (0 = *no*, 1 = *yes*), and dormitory residence status (0 = *no*, 1 = *yes*) were dummy coded. Maternal and paternal education levels were coded from 1 (*uneducated*) to 6 (*master’s degree and above*). Mother and father working outside the home per year were coded from 1 (*never*) to 5 (*more than 6 months*). ^*^*p* < 0.05, ^**^*p* < 0.01, ^***^*p* < 0.001.

**Table S2** Multiple mediation model between harsh parenting and anxiety symptoms in adolescents across three different living arrangements.

| **Dependent variables** | **Independent variables** | **Adolescents who lived with their both parents (*N* = 2615)** | |  | **Adolescents who lived only with their mother (*N* = 384)** | |  | **Adolescents who lived only with their father (*N* = 296)** | |
| --- | --- | --- | --- | --- | --- | --- | --- | --- | --- |
|  |  | β | 95% CI |  | β | 95% CI |  | β | 95% CI |
| Self-efficacy |  |  |  |  |  |  |  |  |  |
|  | Maternal HP | −0.219 | [−0.304, −0.136] |  | −0.046 | [−0.212, 0.105] |  | −0.108 | [−0.265, 0.034] |
|  | Paternal HP | −0.006 | [−0.090, 0.083] |  | −0.215 | [−0.354, −0.075] |  | −0.199 | [−0.340, −0.050] |
| School connectedness |  |  |  |  |  |  |  |  |  |
|  | Maternal HP | −0.106 | [−0.195, −0.022] |  | −0.105 | [−0.256, 0.038] |  | −0.242 | [−0.414, −0.102] |
|  | Paternal HP | −0.093 | [−0.178, −0.004] |  | −0.076 | [−0.235, 0.073] |  | −0.009 | [−0.177, 0.162] |
|  | Self-efficacy | 0.541 | [0.497, 0.583] |  | 0.607 | [0.510, 0.693] |  | 0.488 | [0.336, 0.617] |
| Internet addiction |  |  |  |  |  |  |  |  |  |
|  | Maternal HP | 0.211 | [0.130, 0.302] |  | 0.234 | [0.011, 0.443] |  | 0.234 | [0.064, 0.419] |
|  | Paternal HP | 0.149 | [0.063, 0.231] |  | −0.008 | [−0.204, 0.182] |  | 0.104 | [−0.059, 0.268] |
|  | Self-efficacy | −0.265 | [−0.310, −0.222] |  | −0.332 | [−0.441, −0.211] |  | −0.287 | [−0.426, −0.145] |
| Sleep problems |  |  |  |  |  |  |  |  |  |
|  | Maternal HP | 0.238 | [0.137, 0.339] |  | 0.294 | [0.074, 0.488] |  | 0.055 | [−0.097, 0.216] |
|  | Paternal HP | 0.070 | [−0.025, 0.163] |  | 0.039 | [−0.147, 0.220] |  | 0.260 | [0.091, 0.425] |
|  | Self-efficacy | −0.286 | [−0.335, −0.236] |  | −0.283 | [−0.391, −0.162] |  | −0.348 | [−0.468, −0.223] |
| Anxiety |  |  |  |  |  |  |  |  |  |
|  | Maternal HP | 0.065 | [−0.008, 0.137] |  | 0.053 | [-0.084, 0.188] |  | 0.060 | [−0.131, 0.242] |
|  | Paternal HP | 0.117 | [0.048, 0.187] |  | 0.101 | [−0.013, 0.231] |  | 0.132 | [−0.028, 0.306] |
|  | Self-efficacy | −0.030 | [−0.076, 0.019] |  | 0.029 | [−0.076, 0.147] |  | −0.058 | [−0.190, 0.078] |
|  | School connectedness | −0.253 | [−0.317, −0.195] |  | −0.254 | [−0.394, −0.119] |  | −0.233 | [−0.397, −0.101] |
|  | Internet addiction | 0.213 | [0.166, 0.262] |  | 0.287 | [0.175, 0.409] |  | 0.158 | [0.028, 0.309] |
|  | Sleep problems | 0.347 | [0.292, 0.401] |  | 0.409 | [0.292, 0.535] |  | 0.392 | [0.220, 0.550] |

*Note*. Standardized regression coefficients were reported. Bootstrap sample size = 5000. CI = Confident interval.
